# Fasting during Ramadan is associated with changes in activity and blood glucose excursion in people with type 2 diabetes on three or more anti-hyperglycemic agents PROFAST-3

**DOI:** 10.5339/qmj.2025.76

**Published:** 2025-09-04

**Authors:** Mohammed Bashir, Abdulaziz Al-Homaid, Sabri Boughorbel, Billel Mokeddem, Joao Palotti, Syed Hashim, Ummar Abbas, Tarik Elhadd

**Affiliations:** 1 Endocrine Section, Department of Internal Medicine, Hamad Medical Corporation, Doha, Qatar; 2 Qatar Metabolic Institute, Hamad Medical Corporation, Doha, Qatar; 3 Qatar Computing Research Institute, Hamad Bin Khalifa University, Doha, Qatar; 4 Earkick, Zurich, Switzerland *Correspondence: Mohammed Bashir mbashir@hamad.qa

**Keywords:** Wearable devices, physical activity, sleep, diabetes, Ramadan, blood glucose excursion

## Abstract

**Background:**

The physiological changes during Ramadan in people with type 2 diabetes (T2D) are not well described in the literature. However, advances in technology have created new frontiers to understand these changes. This study aims to understand the impact of Ramadan fasting on blood glucose excursion, vital signs, and physical activities in people with T2D who are on three or more antidiabetic medications.

**Methods:**

This prospective observational study was conducted at Hamad General Hospital, National Diabetes Centre, between February 1, 2020 and May 30, 2020 (covering three months before and including the month of Ramadan). We included people with T2D who were on three or more antidiabetic medications. Medications were adjusted during Ramadan based on international guidelines. Flash glucose monitoring and Fitbit devices were used to monitor glucose levels and physical activity. The primary outcomes were changes in time in range (TIR), time above range (TAR), and time below range (TBR) before and during Ramadan.

**Results:**

We included 18 patients with T2D, of whom 13 were males (72.2%). The mean age was 51.2 years (SD 7.4), the mean HBA1c was 7.8% (SD 1.0), and the mean duration of T2D was 12.5 years (SD 3.1). There were no significant changes in TIR, TAR, and TBR before and after Ramadan. There was no statistically significant difference in the TIR, TAR, and TBR during fasting hours and after iftar. However, the ambulatory glucose profile shows a reduction in glucose levels during fasting hours, reaching a nadir just before iftar, followed by a prolonged period of hyperglycemia post iftar. Physical activity levels decreased during fasting hours but increased approximately one hour before iftar. Multilinear regression analysis showed a positive correlation between engaging in vigorous physical activity and the TBR during fasting hours [*β*-coefficient (95% CI): 0.26 (0.07–0.45), *p* < 0.05].

**Conclusion:**

Our findings show no significant changes in the overall glucose profile, except for prolonged post-iftar hyperglycemia. Intensive physical activity during fasting hours can increase the risk of hypoglycemia. This studyhighlights the need for further in-depth research to better understand the impact of lifestyle changes on blood glucose excursion during Ramadan.

## 1. INTRODUCTION

**Figure 1.**
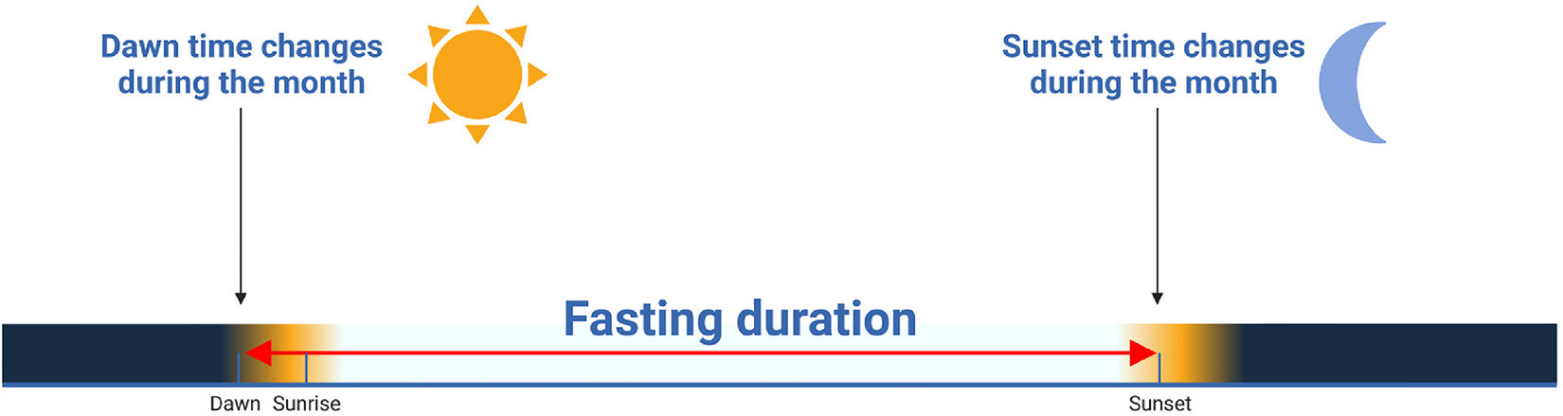
Day representation in the month of Ramadan. Fasting starts at dawn time and ends at sunset time. The time of dawn and sunset varies depending on the time of year. Ramadan occurs in the ninth month of the Islamic calendar, Hijri. Hijri calendar days are 11 days shorter than Gregorian calendar days.

Ramadan is the ninth month of the Islamic Hijri calendar, during which Muslims worldwide observe fasting from dawn until sunset. The global prevalence of type 2 diabetes (T2D) is rising and is projected to continue increasing, particularly in countries with predominantly Muslim populations.^1^ Fasting during Ramadan presents unique challenges for individuals with T2D. During Ramadan, factors such as fasting duration, temperature, working hours in some countries, physical activities, and sleep patterns affect the fasting experience. For instance, in 2021, the fasting hours ranged from 11.5 hours in Australia to 20 hours in Norway. These modulating factors have significant implications on the body’s energy hemostasis, with even more complex effects in individuals with T2D. Moreover, T2D is a heterogeneous condition characterized by multiple phenotypes (Figure 1).^2^

Moreover, the recent availability of multiple antidiabetic medications has expanded therapeutic options and endless possibilities for combination therapies. The heterogeneous fasting conditions, the heterogeneity of T2D, and the endless possibilities of antidiabetic combinations contribute to the complexity of managing diabetes during Ramadan. Although fasting is not obligatory for individuals with chronic conditions such as diabetes, many patients with T2D still choose to fast.^3^ Therefore, patients with T2D must consult their physicians before deciding to fast.

Recent advances in wearable technology that captures physiological features have gained popularity in the medical field. Data from these devices have provided rich and complementary information to what is available in electronic health records (EHR). Even though EHR data is recorded by qualified clinical staff, it tends to be sparse and does not fully reflect the subject’s overall health status. In contrast, wearable devices continuously monitor physiological data and can provide important details about changes in health status. These recorded data can aid in monitoring chronic diseases and rationalize clinical care delivery.^4^ The availability of wearable data has enabled the development of new methods for predicting and analyzing health conditions, such as hypoglycemia.^5^ Subjects with chronic conditions like diabetes could benefit from wearable technologies such as smartwatches and flash glucose monitors (FGMs). Smartwatches provide essential information on physical activity, heart rate, and sleep, while FGM continuously monitors blood glucose levels. By recording synchronized data from both devices, it is possible to investigate the influence of physical activity and sleeping patterns on the glucose levels.

Given the complexity of diabetes and fasting conditions, it is essential to understand the changes in blood glucose excursions that occur during Ramadan by incorporating as many relevant variables as possible. Furthermore, during Ramadan, both the frequency and quantity of meals change significantly. Almost all fasting Muslims eat a meal known as iftar (sunset meal) at the same time after sunset, and another meal known as suhur (pre-dawn meal) is consumed 1–2 hours before dawn. Some individuals tend to reduce their physical activities during fasting hours, while others may choose to exercise an hour or two before iftar. This study aims to examine the impact of physical activity on glucose parameters before and during Ramadan in patients with T2D who are treated with three or more antidiabetic medications. We aim to combine data from two wearable devices: FGM Freestyle Libre (Abbott Diagnostics) for glucose measurements, and the Fitbit-2 pedometer for monitoring physical activity. In Qatar, Ramadan in 2020 began on April 23 and lasted for 30 days. The fasting hours were approximately 14.5 hours per day, with average daytime temperatures ranging from 35^o^C to 38^o^C.

## 2. METHODS

### 2.1. Data set

The PROspective PROFAST Ramadan Study (PROFAST-3) is a prospective longitudinal study designed to assess the physiological and glycemic changes in patients with T2D who are treated with three or more antidiabetic agents and who choose to fast during the month of Ramadan, using wearable devices. The inclusion criteria were similar to those of the two previous PROFAS studies and included: (i) adults patients aged 18–79 years with T2D and on a stable treatment with three or more antidiabetic medications that include either a sulphonylurea (SU) or insulin, planning to fast during the month of Ramadan; (ii) glycated hemoglobin (HBA1c) ≤13.0%; and (iii) estimated glomerular filtration rate >30 ml/min. Exclusion criteria included: (i) a history of recurrent hypoglycemia (more than two episodes of symptomatic hypoglycemia per week) or hypoglycemia unawareness; (ii) admission with more than two episodes of diabetic ketoacidosis (DKA) or with hyperosmolar nonketotic coma in the preceding year or with an episode of DKA within the previous three months before the start of Ramadan; (iii) active coronary artery disease, congestive cardiac failure, advanced co-morbidities, advanced diabetes microvascular complications; and (iv) newly diagnosed cancer or those undergoing cancer treatment.^6,7^ In 2020, the fasting duration was approximately 15 hours, with iftar around 6:20 PM and suhur around 3:30 AM.

We analyzed the data collected from 18 subjects who participated in a study conducted in 2020. The patients were interviewed at two time points: 4–6 weeks before Ramadan and 2–3 weeks after Ramadan began. During the initial visit, the patients received instructions on dietary modification and exercise, along with adjustments to their antidiabetic medications, based on the PROFAST Ramadan study protocol.^6^ We used FGM Freestyle Libre (Abbott Diagnostics) to capture glucose data for two weeks at the two time points, and the libre-link online application to access the recorded data. The patients concurrently wore a Fitbit-2 pedometer, which recorded their activity at 15-minute intervals. Data collected included step count, average heart rate, physical activity intensity, timing, and staging. Pre-Ramadan medication adjustments included a 50% reduction in SU, a maximum metformin dose of 1 g, no changes to dipeptidyl peptidase-4- inhibitors (DPP4i) or sodium glucose transporters-2 inhibitors (SGLT2i), a 30–50% reduction in insulin, and no change to glucagon like peptide-1 (GLP-1). Patients were advised to break their fast if they developed hypoglycemia during fasting.^6,7^

### 2.2. Tools

We performed the statistical analysis using Python 3.9. Categorical variables were expressed as percentages (%), while continuous variables were reported as mean ± standard deviation or median with interquartile range, as appropriate. A paired t-test was used to compare the time in range (TIR) values before and during Ramadan, as well as between fasting and non-fasting periods during Ramadan. Linear regression analyses were conducted to model the odds or likelihood of remaining within TIR, time below range (TBR), and time above range (TAR). Variables with low variability were removed using a standard deviation threshold of 0.001. Highly correlated variables with a correlation threshold of 0.8 were also excluded. We used the scikit-learn module (version 1.0.2) to normalize the data to zero mean and unit variance for the regression analysis. The Statsmodel (version 0.13.2) module was employed for the linear regression analysis. A purposeful selection method was used to build the models across the Pre-Ramadan, Ramadan, and fasting periods. The ggplot function in R was used to plot the 15-minute percentile values for continuous glucose monitoring (CGM), heart rate, steps, TIR, TBR, and TAR. The matplotlib library in Python was used to generate the data availability plot.

To build the models, we used the OLS (ordinary least squares) function from statsmodels.regression.linear_model with the default parameters.

### 2.3. Outcomes

The primary outcomes of this study were the TAR, TIR, and TBR, measured before and during Ramadan. The secondary outcome was to identify the most influential factors affecting glucose changes. The factors assessed included the number of steps, exercise METs (metabolic equivalents), and heart rate; the impact of exercise on blood glucose excursion; the differential impact of exercise before and after iftar on blood glucose excursion during Ramadan; the changes in sleeping patterns; and the impact of these sleep changes on blood glucose excursion during Ramadan.

### 2.4. Ethical statement

The Institutional Review Board of Hamad Medical Corporation, Doha, Qatar, has approved this study (MRC reference number 16437-16). All subjects signed an informed consent before enrollment in the study. The study was conducted in accordance with the declaration of Helsinki.

### 2.5. Data preprocessing and statistical analysis

Fitbit and Freestyle Libre data were collected from all participants over a three-month period, from February 2020 to the end of May 2020. The data were stored on the System for Integrating Health Analytics (SIHA), a cloud-based platform that integrates clinical data with data from wearable devices.^8^ SIHA standardizes the data formats across various devices, supports a wide range of wearables, harmonizes the data across devices, and facilitates clinical workflows. We extracted intraday longitudinal data for heart rate, calories, steps, distance, sleep, and CGM. All features were resampled to 15-minute intervals. TAR was defined as time spent with glucose levels >180 mg/dl; TIR as time spent between 70 and 180 mg/dl, and TBR as time spent <70 mg/dl.^9^ The dataset was filtered so that each patient had at least three consecutive days with a minimum of 2,000 steps per day. Step counts were treated as continuous variables.

Baseline data were collected for HBA1c, triglycerides (TGs), high-density lipoprotein (HDL), low-density lipoprotein (LDL), gender, age, weight, and body mass index (BMI). Data aggregation was done daily for both – the glucose and the physical activity data – to address the missing data in both modalities (CGM and wearable), and to enhance the alignment between the two modalities. Also, the aggregated data captured the latency in glucose changes better than the fine-grained time-stamped data – for instance, CGM readings typically peaked around two hours after sunset during iftar. In addition, aggregated data helped account for unmeasured variables such as meal intake. The association between glucose levels and physical activity on a daily basis would account for the effects of meals on glucose.

The aggregation was performed at a daily level for each patient’s static features. Variable features were grouped by patient ID, Ramadan status, and date, applying a sum operation and then dividing by 96 – corresponding to 96-fifteen-minute blocks daily. This aims to account for extreme values if the patient has a missing number of time stamps in a day. After the group-by operation, we merged the data again using patient’s ID, Ramadan status, and date. Next, based on domain knowledge, we selected relevant features and removed irrelevant columns and columns with missing values for each experiment. In addition, we used drug categories in the analysis. To account for bias before and during Ramadan, we made the patients have the same number of instances in both periods, which means we removed the patients who had no instance before or during Ramadan. After separating the dataset into X and Y, we removed columns with a correlation >0.95 following all the preprocessing steps described above. We ended up with the features displayed in Supplementary Figures 1 and 2.

**Figure 2.**
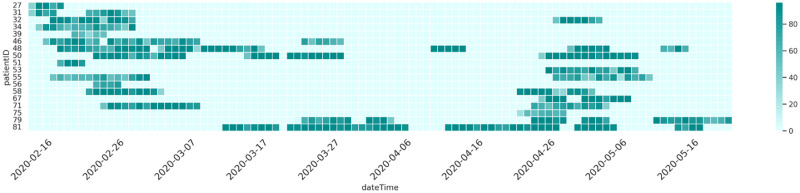
Summary of the wearable device data. CGM and smartwatch data are resampled at 15-minute intervals and synchronized. The heatmap shows daily data availability (0%=fully missing, 100%=complete). Missing data primarily stems from manual CGM-phone synchronization. All subjects have sufficient representative days before and during Ramadan. Data availability for the 18 PROFAST Ramadan Study 2020 participants. Ramadan 2020 was observed from April 23, 2020 to May 23, 2020. Maximum value of a cell is 96.

Figure 2 provides a visual summary of the data collected from the wearable devices. The CGM and the smartwatch data were resampled at 15-minute intervals and time-synchronized. The heatmap indicates the percentage of available data for each study day with 0% indicating a complete absence of data and 100% indicating no missing data. The data missingness is mainly caused by the CGM devices, as synchronization with the mobile phone must be done manually. Each subject has sufficient representative days from both periods, before and during Ramadan.

## 3. RESULTS

We recruited 18 subjects, comprising 13 males and 5 females. As shown in Table 1, the mean age was 51.2 years, the mean weight was 77.1 kg, the mean BMI was 29.1 kg/m^2^, the mean duration of diabetes was 12.5 years, the mean HBA1c was 7.8%, and the mean LDL was 2.7 mmol/l. Most patients were on metformin (83%), 50% were on basal insulin, and 16% were on SU.

**Figure 3.**
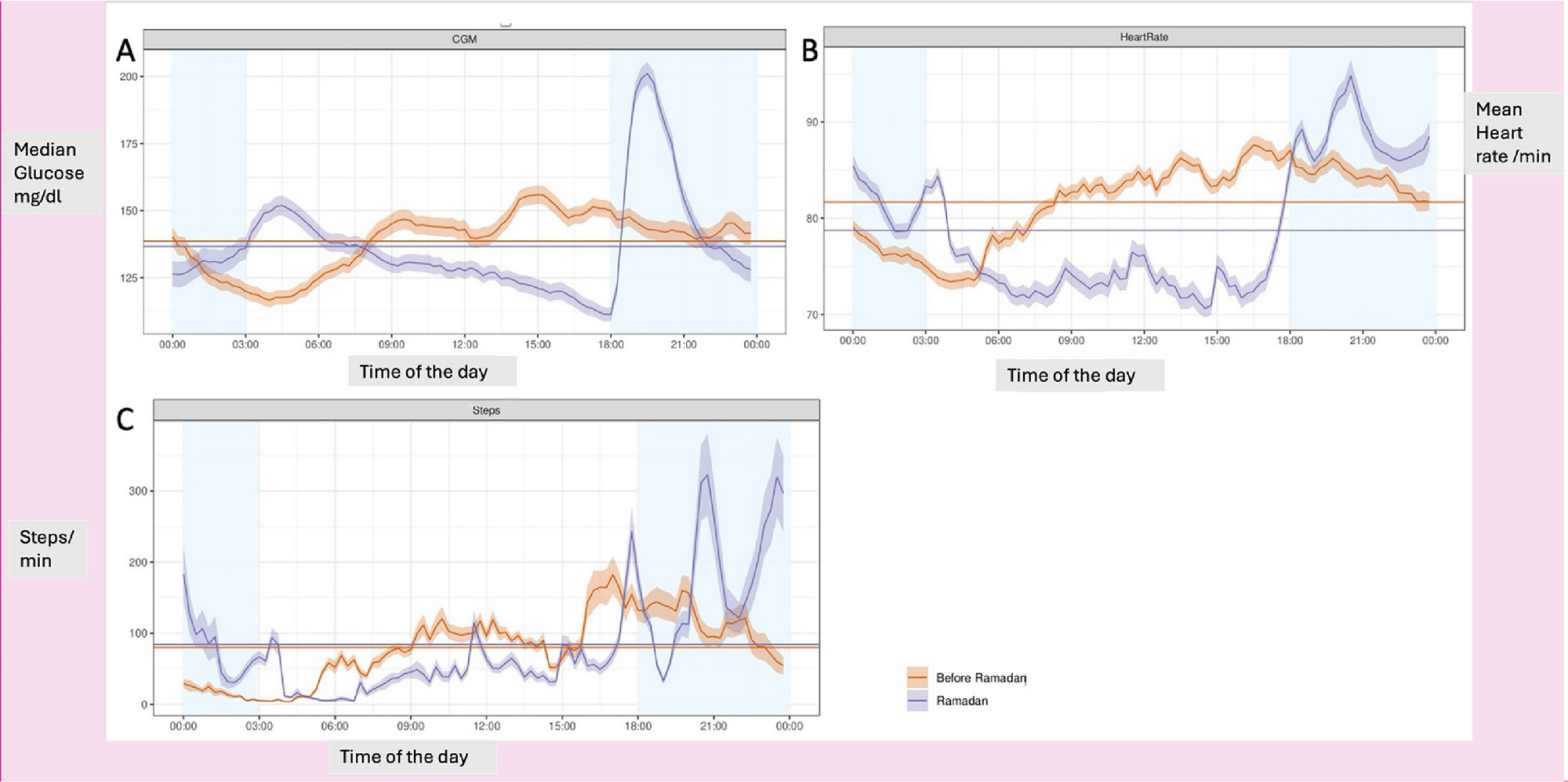
Physiological changes during Ramadan. (A) Median glucose levels (mg/dl). (B) Mean heart rate/min. (C) Mean steps/15 min.

Figure 3 shows the changes in physiological parameters and blood glucose excursion during and after Ramadan. Figure 3A shows a gradual decline in median glucose levels during fasting, with a nadir just before iftar followed by a significant spike that lasts approximately 4–5 hours after iftar and another smaller spike after suhur. Figures 3B and C show the mean heart rate/min and mean steps/15 min during and after Ramadan. As shown in the graph, both the number of steps and heart rates tend to be lower during the fasting hours, increase within the hour before iftar, and continue to rise afterward. We have identified two peaks of increased activities, the hours before iftar and three hours after iftar.

**Figure 4.**
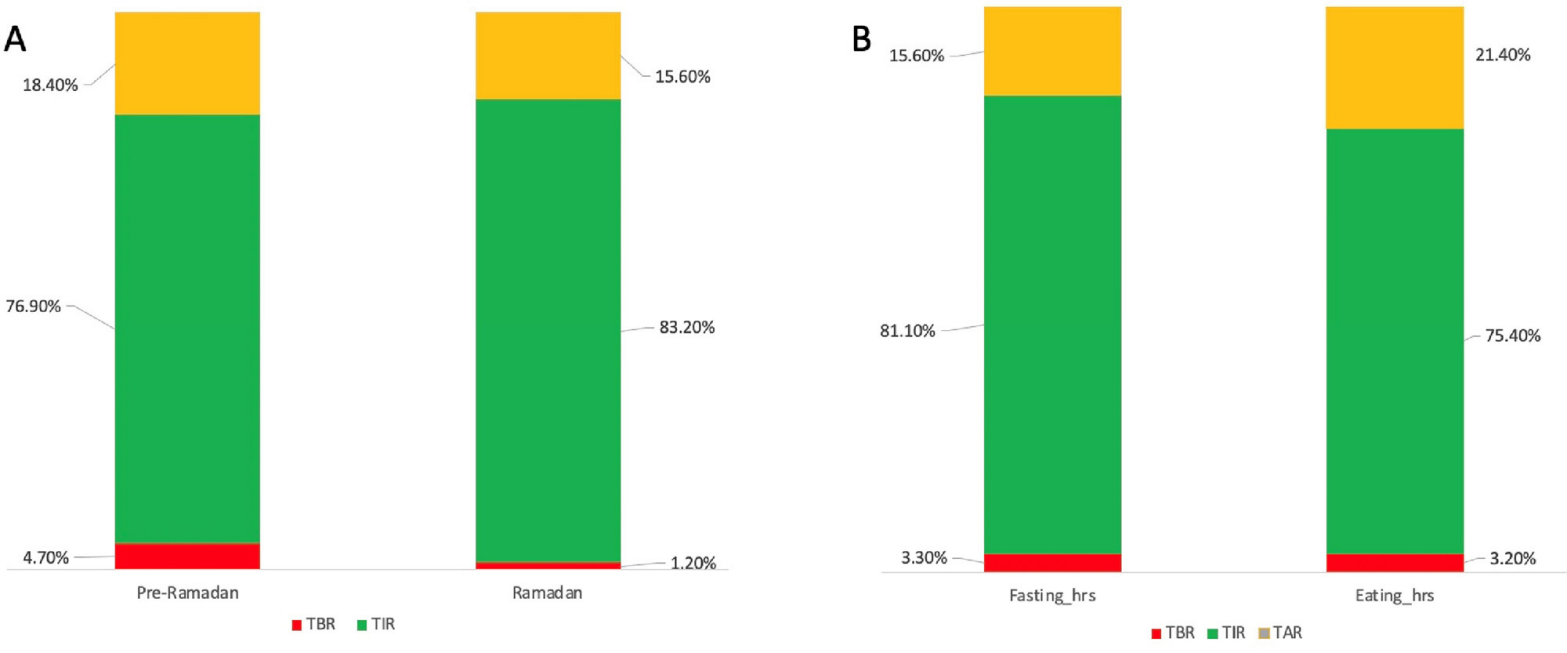
Changes in glucose parameters (A) before and after Ramadan and (B) during fasting hours and after iftar. TAR: Time above range, TIR: Time in range, TBR: Time below range.

Figure 4 shows the ambulatory glucose parameters before and during Ramadan. As shown in Figure 4A, there was no significant difference between TAR (18.4% vs. 15.6%, *p* = 0.69), TIR (76.9% vs. 83.2%, *p* = 0.83), and TBR (4.7% vs. 1.1%, *p* = 0.53) in pre-Ramadan and during Ramadan. As shown in Figure 4B, there was no significant difference between the ambulatory glucose profile (AGP) parameters during fasting hours and after iftar: TAR (15.6% vs. 21.4%, *p* = 0.74), TIR (81.1% vs. 74.4%, *p* = 0.84), and TBR (3.3% vs. 3.2%, *p* = 0.58). Multivariate linear regression analysis (Table 2) showed that vigorous exercise was associated with increased TBR during fasting hours [β-coefficient (95% CI): 0.26 (0.07–0.45), *p* < 0.05].

## 4. DISCUSSION

This is the first study to objectively describe the changes in physical activities and blood glucose excursions during Ramadan in patients with T2D. The study cohort had a relatively well-controlled glycemic parameter with an average HBA1c of 7.8% and TIR of 76.9%. The Fitbit data showed a general slowdown in activities during the fasting hours of Ramadan. There were two spikes in the activity levels: one an hour before iftar and another three hours after iftar. Data on blood glucose excursion showed a continuous decline in the average glucose levels during the fasting hours with the nadir at iftar time. Following iftar, there was a spike in the average glucose level that lasted almost six hours, followed by a smaller spike after suhur that lasted approximately three hours. We have seen no significant changes in the ambulatory glucose parameters before and during Ramadan. Numerically, hyperglycemia was more frequent after iftar than during fasting hours. There was no difference in TBR before and during Ramadan, or before and after iftar.

These findings provide critical information that can help people with T2D fast safely during Ramadan. Regression analysis showed a correlation between both the degree and intensity of activities during the fasting hours and the TBR. We have previously shown that patients with physically active occupations have a higher risk of asymptomatic hypoglycemia than the sedentary groups.^10^ This study provides more evidence to support the critical role that physical activity should play when drafting medication adjustment plans during Ramadan. The diabetes and Ramadan (DAR) guidelines recommend reducing the basal insulin dose by 15–30% during Ramadan.^11^ This reduction might not be optimal for patients who remain highly active during fasting hours. Furthermore, the guidelines recommend reducing SU doses in patients with adequate glycemic control.^11^ However, it might be necessary to halve the SU dose in individuals who are physically active during fasting hours – regardless of glycemic control. Lastly, active patients should monitor their glucose levels more frequently during fasting hours.

The increase in physical activity during the hour before iftar is worrisome. There is an increase in the number of people exercising during this hour, hoping to induce more weight loss. The AGP showed a continuous decline in average glucose levels, reaching its nadir at iftar. Patients with T2D, particularly those on insulin or SU, are more vulnerable to hypoglycemia during this time of the day. Furthermore, symptoms of hypoglycemia are hard to distinguish from the general tiredness and hunger associated with fasting.^10^ Besides, ketone production increases during the last few hours of fasting.^12^ Hence, educating patients with T2D to avoid exercise during this time of the day is essential.

Despite adequate glycemic control, we still observed a significant spike in glucose levels that spans over six hours after iftar. Another shorter spike is seen after suhur. This indicates an imbalance in the carbohydrate consumption. Both the quality and the quantity of carbohydrate intake are altered during Ramadan. Alzhrani et al. showed that the total daily calorie intake during Ramadan in Saudia Arabia is increased by approximately 10%, and the total daily carbohydrate intake is increased by approximately 12%.^13^ A study from Turkey showed an increase in the consumption of unhealthy food during Ramadan.^14^ Consuming these high levels of carbohydrates over two meals is associated with a poorer glycemic profile. Hakeem et al. examined 1,950 daily food records during Ramadan and found an increase in higher energy intake and poor distribution of carbohydrates.^15^ Furthermore, the study showed that consuming four small meals is associated with better glycemic control.^15^ Hence, three critical changes might be necessary: reduction in carbohydrate intake, distribution of carbohydrate intake over more than two meals, and the increase or the introduction of bolus insulin with iftar.

The small sample size limits our findings. We also lack data on food intake during iftar and suhur and socio-economic demographics that affect food choices. The study’s main strength is its objective overview of physiological and behavioral changes during Ramadan from multiple data sources.

## 5. CONCLUSION

Fasting during Ramadan is associated with multiple changes in physical activities and blood glucose excursion. Understanding these changes is crucial for helping patients with T2D who are on three or more antidiabetic medications to fast safely. We observed no changes in the overall AGP; however, significant changes were noted in postprandial blood glucose excursions during Ramadan. We have also shown a positive correlation between the intensity of activities during the fasting hours and the TBR. While patients with T2D should be encouraged to remain active, increased physical activities just before iftar should be avoided due to the increased risk of hypoglycemia.

## ACKNOWLEDGMENTS

The authors would like to thank patients who have participated in this study. We would like to acknowledge all the physicians who are part of the PROFAST program. We are greatly indebted to Abbott Diagnostics for their generous donation of the Freestyle Libre devices.

## AUTHORS’ CONTRIBUTIONS

TE and MB: conceptualization, methodology and data collection. MB and AH: critical review and revisions. AH, SB, BM, JP, SH, UA, and MB: data analysis and data interpretation. All authors: literature review, manuscript writing, and review and approval of the final manuscript.

## DATA AVAILABILITY STATEMENT

Data is available from the corresponding author upon reasonable request.

## COMPETING INTERESTS

The authors have no conflicts of interest to declare.

## Supplementary Material

Supplementary Figure 1. CGM horizon plots. >250, 220–250, 180–200, 70–180, 54–69, <54.

Supplementary Figure 2. Steps horizon plots.

## Figures and Tables

**Table 1.** Baselines characteristics of the study cohort, including demographics, anthropometrics, metabolic data, and medications.

Baseline characteristics	
Gender	Males (13) Females (5)
Age (mean ± SD)	51.2 years (7.4)
Weight (mean ± SD)	77.1 kg (11.4)
BMI (mean ± SD)	29.1 kg/m^2^ (3.0)
Duration of diabetes (mean ± SD)	12.5 years (3.1)
HBA1c (mean ± SD)	7.8% (1.0)
LDL (mean ± SD)	2.7 mmol/l (0.4)
HDL (mean ± SD)	1.0 mmol/l (0.3)
TGs (mean ± SD)	2.1 mmol/l (2.1)
Metformin	83%
SU	16.6%
DPP4i	77.7%
SGLT2i	72.2%
GLP-1	11.1%
Pioglitazone	27.7%
Basal insulin	50%
Bolus insulin proportion	11.1%

SU: Sulphonylurea, DPP4i: Dipeptidyl peptidase inhibitors, GLP: Glucagon like peptide-1.

**Table 2.** Linear regression analysis for predicting the percentage of time below range (TBR) in the ambulatory glucose profile.

	TBR (before Ramadan) [β-coefficient (95% CI)]	TBR (during Ramadan) [β-coefficient (95% CI)]	TBR (during Ramadan fasting hours) [β-coefficient (95% CI)]
Light activity		−0.03 (−0.15 to 0.08)	0.21 (−0.4 to 0.81)
Sedentary activity	0.18 (0.05 to 0.31)*		0.25 (−0.45to 0.96)
Steps (per minutes)	0.01 (−0.15 to 0.16)	−0.32 (−0.53 to -0.1)*	0.11 (−0.36 to 0.14)
Vigorous activity	−0.18 (−0.29 to −0.07)*		0.26 (0.07 to 0.45)*
METs		0.52 (0.09 to 0.94)*	0.08 (−0.63 to 0.78)

Corrected for age, weight, HBA1c, creatinine, metformin, pioglitasone, DPP4i, SGLT2i, basal insulin, and bolus insulin: *, corrected for sleep.
