# Supplementary figures and images for: Fasting during Ramadan is associated with changes in activity and blood glucose excursion in people with type 2 diabetes on three or more anti-hyperglycemic agents PROFAST-3
Source: Qatar Med J. 2025 Sep 4;2025(3):76. doi: 10.5339/qmj.2025.76 (PMC12439493; doi:10.5339/qmj.2025.76)

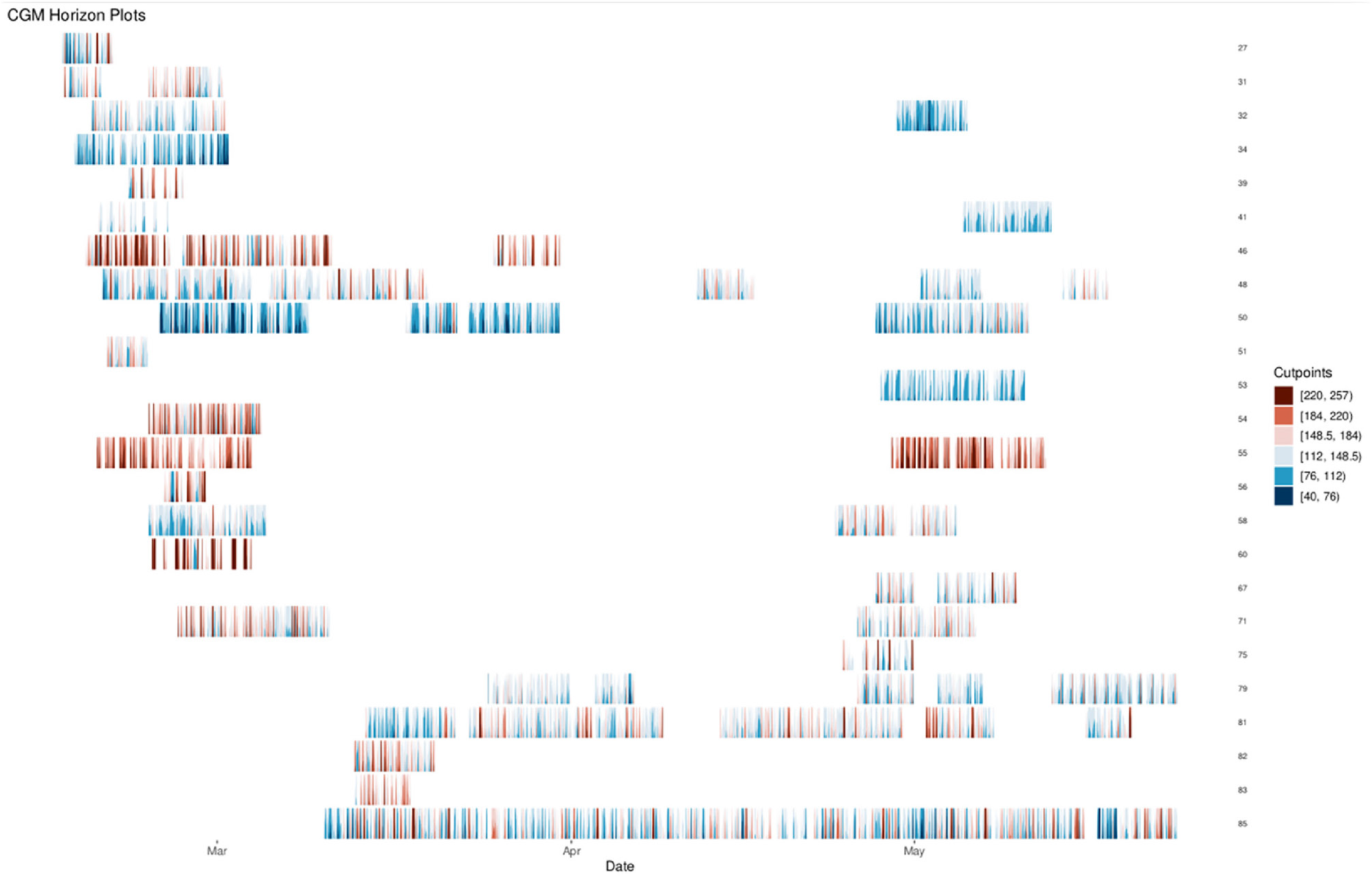

Supplement: Supplementary Figure 1. — CGM horizon plots. >250, 220–250, 180–200, 70–180, 54–69, <54. [file qmj-2025-03-076-g005.tif]

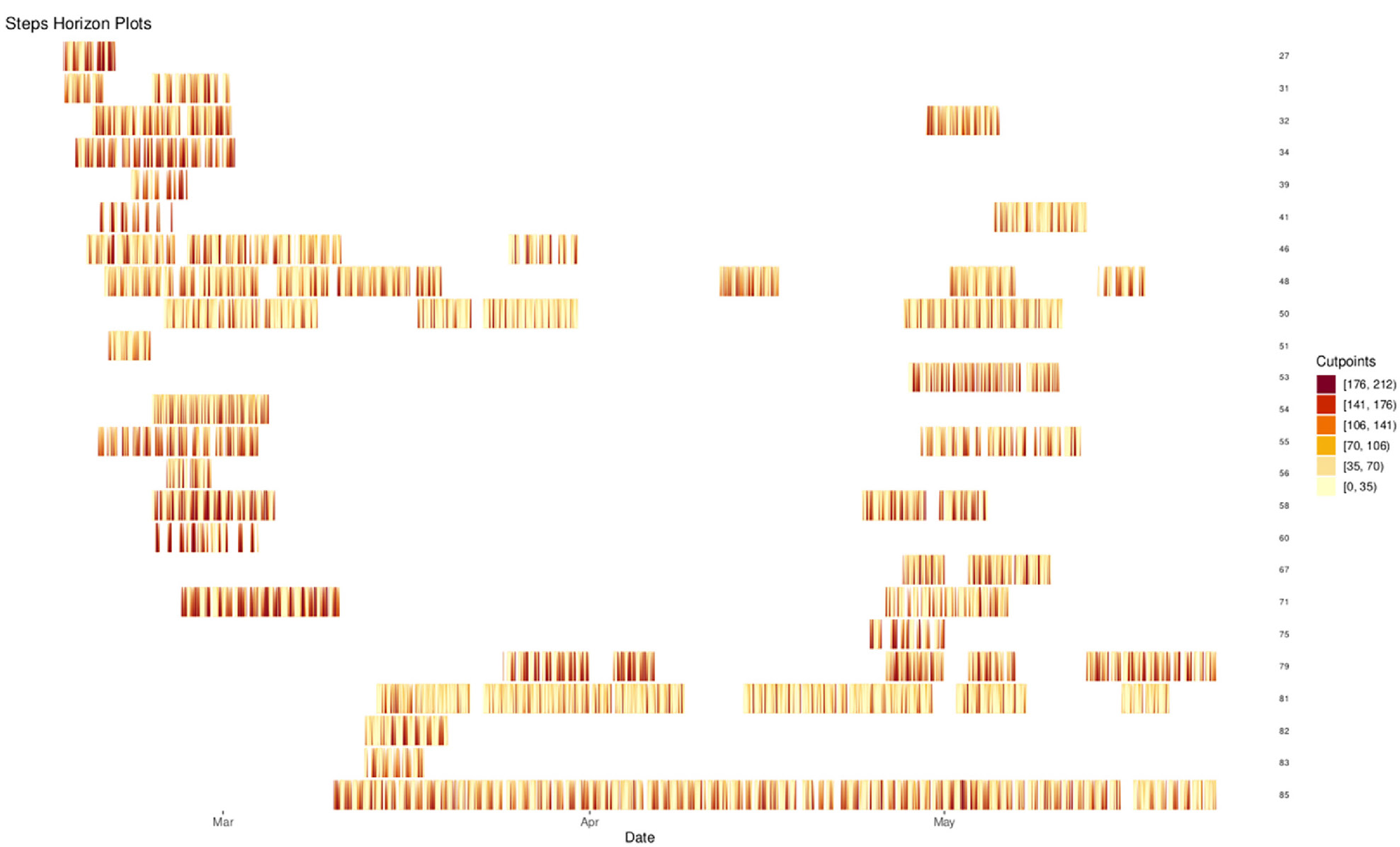

Supplement: Supplementary Figure 2. — Steps horizon plots. [file qmj-2025-03-076-g006.tif]
